# ESTs from a wild *Arachis *species for gene discovery and marker development

**DOI:** 10.1186/1471-2229-7-7

**Published:** 2007-02-15

**Authors:** Karina Proite, Soraya CM Leal-Bertioli, David J Bertioli, Márcio C Moretzsohn, Felipe R da Silva, Natalia F Martins, Patrícia M Guimarães

**Affiliations:** 1 Departamento de Biologia Celular, Universidade de Brasília, Campus I, Brasília, DF. Brazil; 2 EMBRAPA Recursos Genéticos e Biotecnologia. Parque Estação Biológica, CP 02372. Final W5 Norte, Brasília, DF. Brazil; 3 Universidade Católica de Brasília, Pós Graduação Campus II, SGAN 916, Brasília, DF. Brazil

## Abstract

**Background:**

Due to its origin, peanut has a very narrow genetic background. Wild relatives can be a source of genetic variability for cultivated peanut. In this study, the transcriptome of the wild species *Arachis stenosperma *accession V10309 was analyzed.

**Results:**

ESTs were produced from four cDNA libraries of RNAs extracted from leaves and roots of *A. stenosperma*. Randomly selected cDNA clones were sequenced to generate 8,785 ESTs, of which 6,264 (71.3%) had high quality, with 3,500 clusters: 963 contigs and 2537 singlets. Only 55.9% matched homologous sequences of known genes. ESTs were classified into 23 different categories according to putative protein functions. Numerous sequences related to disease resistance, drought tolerance and human health were identified. Two hundred and six microsatellites were found and markers have been developed for 188 of these. The microsatellite profile was analyzed and compared to other transcribed and genomic sequence data.

**Conclusion:**

This is, to date, the first report on the analysis of transcriptome of a wild relative of peanut. The ESTs produced in this study are a valuable resource for gene discovery, the characterization of new wild alleles, and for marker development. The ESTs were released in the [GenBank:EH041934 to EH048197].

## Background

Peanut or groundnut (*Arachis hypogaea *L.) is the fourth most important oil seed in the world, cultivated mainly in tropical, subtropical and warm temperate climates [1]. It is an important crop for both human and animal food. Its yields are reduced around the world by diseases including fungal leaf-spots caused by *Cercospora arachidicola *[Hori] and *Phaseoisariopsis personata *[Berk. & MA Curtis], the rust *Puccinia arachidis *[Speg.], groundnut rosette disease, and root-knot nematodes (*Meloidogyne *ssp.), the later causing losses of up to 12% in United States and India [2]. High salinity and drought are also important reducers of yield in many parts of the world.

Wild relatives are an important source of genes for resistances to biotic and abiotic stresses that affect crop species. The genus *Arachis *arose in South America and its approximately 80 species have adapted to a wide range of environments. The cultigen *A. hypogaea *probably arose from a single or few events of hybridization involving AA and BB genome species. The hybrid underwent spontaneous duplication of chromosomes to produce the allotetraploid *A. hypogaea *with genome type AABB [3]. This difference in ploidy rendered peanut sexually isolated, giving this species a very narrow genetic basis [4,5].

Due to this sexual isolation, the introgression of wild genes is only possible through complex crosses or genetic transformation. To date, there is only one case of successful introgression of genes from wild species into *A. hypogaea *to produce commercial cultivars of peanut [3]. This was through the use of a synthetic allotetraploid (also called a synthetic amphidiploid, or amphiploid), created by crosses between wild *Arachis *species. Although the wild species used were non-ancestral, the crosses, in some ways, approximate a re-synthesis of the species *A. hypogaea*. Genetic transformation of peanut, although difficult, has also been accomplished by a number of techniques [6-10].

For improvement of the peanut crop, there is a need to both identify novel genes with potential agronomic interest and to either develop molecular markers associated with such genes for use in marker assisted selection, or to use genes in genetic transformation. EST sequencing projects have been contributing to gene discovery and marker development as well as shedding light on the complexities of gene expression patterns and functions of transcripts [11-13].

A few projects on the generation of ESTs from *A. hypogaea *have recently been accomplished, using different tissues and conditions: plants subjected to *Aspergillus parasiticus *infection and drought stress [14], late leaf spot [15] and unstressed tissues [16]. However, at present a total of roughly 25,000 *Arachis *ESTs are available in Genbank, all derived from cultivated peanut *A. hypogaea *and none from wild species of *Arachis*.

*Arachis stenosperma *is a wild diploid species which presents a number of disease resistances. Plants of this species form fertile hybrids with *A. duranensis *[17] (the AA genome donor of peanut [18,19], and is therefore a potential AA genome donor for synthetic allotetraploids. It is also a parent for the population from which was derived the only SSR-based map of *Arachis *[17].

Here we report the partial sequences, database comparisons and functional categorization of 8,785 randomly collected cDNA clones of *A. stenosperma *and their use for the development of 107 microsatellite markers. These data will be useful for those searching for novel genes from wild *Arachis*.

## Results

### cDNA libraries construction, sequencing and ESTs analysis

Four cDNA libraries were constructed, one from bulked root samples collected at 2, 6 and 10 days after inoculation with *Meloidogyne arenaria *race 1, one from roots inoculated with *Bradyrhizobium japonicus*, another from non -inoculated and a fourth from healthy leaves. From the initial plating, the libraries were estimated to contain 10^7 ^pfu/mL (plaque- forming units) (non-inoculated roots) and 10^8 ^pfu/mL (inoculated roots) and 10^9 ^pfu/mL (healthy leaves). The insert size of 48 randomly picked clones ranged from c. 400 to 1500 bp, with an average of c. 550 bp. From the 8,785 clones, 2,520 were discarded by the trimming procedure. Forty three (0.5%) clones represented ribosomal sequences, 1,033 (11.8%) had sequence slippage, and 1,444 (16.5%) were too small or had too low quality to be incorporated into the analysis. The 6,265 (71.3%) cleaned reads were assembled in 3,500 clusters, being 963 contigs and 2,537 singletons [GenBank:EH041934 to EH048197]. Of the 3,500 clusters analysed, 44.1% did not match genes of known functions. Table 1 summarizes this data. The most abundant reads and their Blast homologies are described in Table 2. From these 3,500 unique sequences only 502 are similar to the *A. hypogaea *ESTs already deposited in GenBank (Blastn <e^-30^). Only 161 code for proteins that are similar to those already described for *Arachis *(Blastx value <e^-10^).

**Figure 1:**
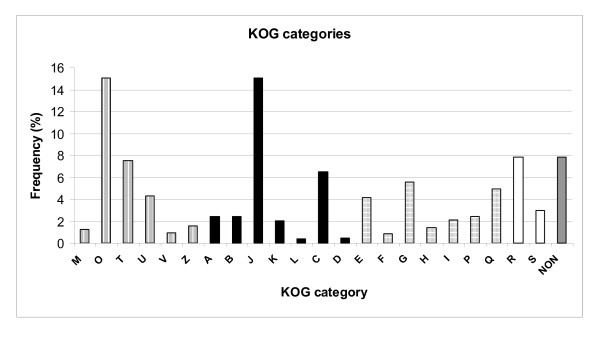
Functional classifications and comparative analysis of the ESTs of *A. stenosperma *roots. The ESTs were classified on the basis of their biological functions by alignment to proteins of the Genbank. Bars with vertical stripes represent frequency of sequences with homology with genes involved in cellular processes and signaling, black bars, information storage and processing, bars with horizontal stripes, metabolism, white bars, poorly characterized ESTs and grey bar, non-conclusively classified ESTs (that showed homology with at least two categories, so they were grouped separately). CELLULAR PROCESSES AND SIGNALING M Cell wall/membrane/envelope biogenesis O Posttranslational modification, protein turnover, chaperones T Signal transduction mechanisms U Intracellular trafficking, secretion, and vesicular transport V Defense mechanisms Z Cytoskeleton INFORMATION STORAGE AND PROCESSING A RNA processing and modification B Chromatin structure and dynamics J Translation, ribosomal structure and biogenesis K Transcription L Replication, recombination and repair METABOLISM C Energy production and conversion D Cell cycle control, cell division, chromosome partitioning E Amino acid transport and metabolism F Nucleotide transport and metabolism G Carbohydrate transport and metabolism H Coenzyme transport and metabolism I Lipid transport and metabolism P Inorganic ion transport and metabolism Q Secondary metabolites biosynthesis, transport and catabolism POORLY CHARACTERIZED R General function prediction only S Function unknown

The annotation of the *A. hypogaea *ESTs was based on sequence homology. Each EST set inherited the annotation form the best match found in BlastX alignment against protein databases at NCBI. On the basis of the KOG (Clusters of Eukaryotic Orthologous Groups of Proteins), the EST sequences in the cDNA libraries were further functionally classified by sorting into 23 putative functional groups (Figure 1).

Protein sequences derived from hypothetical translations of the 3,500 unique sequences are homologous to many classes of proteins. Automatic classification revealed, the main groups of ESTs are related to: cellular processes and signaling, especially those related to post-translational modifications, protein turnover and chaperones (30.6% of all reads); information storage and processing, including various protein kinases (29.3%), and metabolism and energy conversion and sugar, water and ion transporters (21.5%). One drawback of functional classification is the crude approach since the assignments are based on several sets of known proteins and a large percentage of ESTs (7.8%) remained unclassified.

More specifically, sequences of agronomical and medical interest were also found. Sequence contigs related to stress induced genes were numerous and included resistance gene-analogues (RGAs, 35 contigs), pathogenesis-related (PR) proteins (26 contigs), lectins (20 contigs), drought-induced proteins (13 contigs), heat-shock proteins (11 contigs) and aluminium-induced proteins (eight contigs). In addition, there are ESTs whose derived proteins are of potential importance to human health. For instance, homologs to genes encoding allergenicity-related proteins (32 contigs), enzymes involved in the synthesis of isoflavonoids: phenylalanine ammonia-lyase (two contigs), resveratrol synthase and stilbene synthase (15 contigs); oxysterol-binding protein (one contig) and tumor suppressor protein (three) were found. Other sequences of interest were related to nodulation (30 contigs) and homologous to retroelements (nine contigs).

The most frequent clones sequenced had BLASTx hits to: auxin-repressed protein-like protein (115 reads), Arah8 allergen (69 reads), type 2 metallothionein (60 reads), PR10 protein (56 reads) and cytokinin oxidase-like protein (44 reads) (Table 2).

### Analysis of microsatellites and development of markers

**Figure 2:**
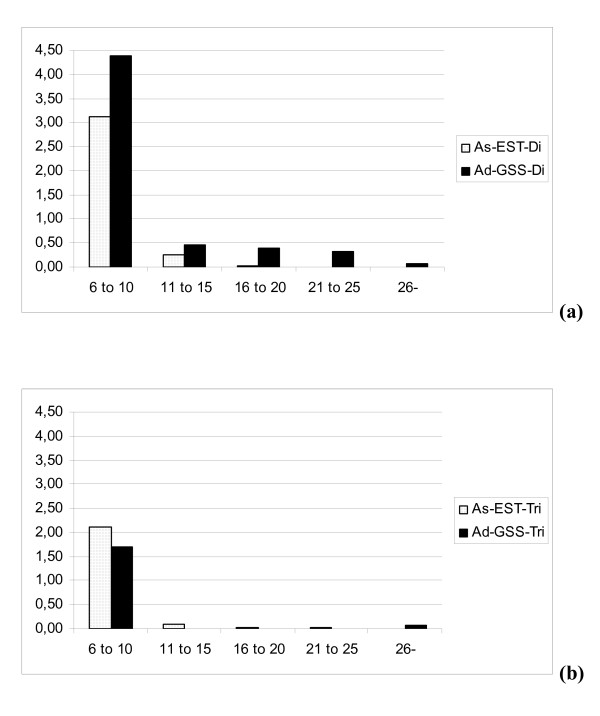
Microsatellite distribution in ESTs from *A. stenosperma *V10309 and Genome Survey Sequences from *A. duranensis*. SSRs were sorted according to motif type and number of repeats. Y axis is percentage of total sequences and X axis is the number of repeats for (A) Di-nucleotide microsatellites and (B) Tri-nucleotide microsatellites.

Out of the 3,500 contig and singleton sequences analysed, 206 (5.9%) had microsatellites. Most of these are di- or tri- nucleotide motifs, being 119 (3.4%) and 79 (2.3%) respectively. The vast majority of the microsatellites (191/206) are short, with 6–10 motif repetitions. Of the di-nucleotide motifs most are TC or AT (102/119). An analysis of *A. hypogaea *clustered transcripts from Genbank gave similar results, except with slightly higher percentages of microsatellite containing sequences (6.8%) and tri-nucleotide repeats (3.4%). In order to compare the microsatellite compositions of non-coding and transcribed genomic sequences in *Arachis *we also analyzed 1,530 clustered *A. duranensis *genome survey sequences (GSSs) from GenBank. *A. duranensis *is a wild species with an AA genome quite closely related to *A. stenosperma*. From these sequences, 118 (7.7%) contained microsatellites, and again the vast majority are di- or tri- nucleotide motifs, being 86 (5.6%) and 27 (1.8%) respectively. As with the EST data, most di-nucleotide microsatellites are TC or AT (70/86). However, there are also some distinct contrasts in the profiles of microsatellites in ESTs compared to genome survey sequences. Di-nucleotide microsatellites of all repeat lengths are more common in genome survey sequences than in ESTs, but tri-nucleotide microsatellites are somewhat more common in the ESTs than the genome survey sequences (Figure 2A and 2B).

From the EST data described in this work, a total of 188 microsatellite markers have been developed and characterized for polymorphism, 81 of these were already published in Moretzsohn *et al*. [17]. From the 107 new ones published here, 84 have been characterized, of these 21 were polymorphic for the AA population, and four for cultivated peanut. Primer sequences, microsatellite types, polymorphism, homologies and linkage groups assigned to the markers are available in Additional File 1.

## Discussion

The most significant stresses of the peanut crop are pathogens and drought. Together with food safety (low levels of aflatoxins and allergenic compounds) they represent the most important targets for crop improvement. Because of the low genetic diversity in the peanut crop, wild relatives are an important source of novel genes. Geographically, *A. stenosperma *is the most widely spread *Arachis *species and, in consequence, has been selected in diverse environments ranging from savannah to coastal dunes. It is sexually compatible with the most probable AA genome donor of cultivated peanut (*A. duranensis*), and therefore is an excellent genome donor candidate for gene introgression. In addition, the species shows signs that it has itself been subject to selection for cultivation traits by South American natives [4]. Therefore, it is a very promising source of new genes for improving cultivated peanut. More specifically, the accession *A. stenosperma *V10309 is very resistant to root-knot nematode, leaf spots and rust fungi (data not shown). For these reasons, *A. stenosperma *V10309 was chosen as the model for this EST project. In this work, a number of clones of agronomic and medical importance were found, and new microsatellite markers were developed and characterized.

### Health-associated genes

Resveratrol-synthase and stilbene synthase are two enzymes involved in the production of resveratrol, a naturally occurring plant compound associated with defense mechanisms against biotic and abiotic stresses [20]. Results from various research studies on edible peanuts have shown that, in humans, resveratrol may protect against atherosclerosis by preventing the oxidation (or breakdown) of the LDL cholesterol in the blood and thus the deposition of cholesterol in the walls of arteries leading to heart disease [21]. It has also been shown to be linked to the suppression of the development of carcinoma cell lines [22]. Chalcone synthase and phenylalanine ammonia-lyase are two key related enzymes involved in the biosyntheses of phytoalexin isoflavonoids in legumes [23]. Isoflavonoids are a class of flavonoids that have estrogen-like activity and which lower serum LDL cholesterol and raise HDL cholesterol, thus having important implications in human health [24]. Oxysterol-binding proteins comprise a large conserved family of cytosolic proteins in eukaryotes. They have been proposed to have a receptor-like role in regulating cholesterol synthesis, being therefore important in the cholesterol metabolism of the human body [25].

In contrast to the potential health benefits of resveratrol and stilbene synthases, allergens in peanut seeds are a major problem. Unexpectedly, the allergen *Ara*H 8 was the second most abundant EST, with 69 occurrences. So far, nine potentially important allergens of peanut have been identified (*Ara*H1 to *Ara*H8 and peanut oleosin) [26]. *Ara*H8 has been described relatively recently; it was deposited in the NCBI in February 2005 from *A. hypogaea*, with a single entry. *Ara*H8 has sequence homology to several pathogenesis-related proteins and may itself be a PR protein. Studies show that allergy to this protein is heavily correlated to allergy to birch pollen [27]. Interestingly, this seemed to be the only allergen expressed abundantly in the roots of *A. stenosperma*.

### Stress and Defense-related genes

Although the plants were kept in the greenhouse, in near-optimum conditions, sequences with hits to genes responsive to biotic and abiotic stresses were found in all four libraries. Similarly, defense-related sequences were previously found in a number of other EST projects with non-inoculated tissue of different species [28,29].

#### RGAs

One mechanism of plant defense, mediated by specific resistance genes, involves the recognition of pathogens by the plant. Among the cellular events that characterize this type of resistance are oxidative burst, cell wall strengthening, induction of defense gene expression, and rapid cell death at the site of the infection [30]. Resistance genes are often organized in clusters, and consequently RGAs have been shown to be genetically linked to known R-genes, or indeed to be fragments of the known R-genes themselves [31-34].

The first published study on RGAs of *Arachis *was by Bertioli *et al*. [35] who isolated 78 complete contigs from *A. hypogaea *and four wild relatives, including *A. stenosperma *V10309, used here. Recently, Yuksel *et al*. [36] isolated 234 RGAs from *A. hypogaea*. In the ESTs produced in this study 35 non-redundant sequences had significant homology to *A. thaliana *NBS containing genes.

#### Auxin-repressed protein

The plant hormone auxin regulates various growth and developmental processes including lateral root formation, apical dominance, tropism and differentiation of vascular tissue [37]. A number of genes have been classified as auxin-response genes, with their expression levels increasing within minutes of auxin application, independent on the *de novo *protein synthesis [38,39]. However, to date, auxin-repressed protein (ARP) genes and their role in plant growth and development are relatively understudied. So far, three orthologs of ARP have been isolated and described: *SAR5 *– isolated from strawberry receptacles and positively correlated with fruit maturation, *PsDRM1*- dormancy related protein from pea and *RpARP*- isolated from the legume tree *Robinia pseudoacacia *(black locust) which is negatively related to hypocotyl elongation [40]. Although its biological function has not yet been clarified, *RpARP *was found to be expressed in various developmental stages and tissues and to play an important role in biological processes that are characteristic under non-growing or stress conditions [40]. In this study, a clone encoding an amino acid sequence with homology to the auxin repressed protein domain (pfam05564.4) was the most expressed sequence in *A. stenosperma *roots (Table 2). The clone's top BLASTx hit was to an auxin repressed protein homolog from *Manihot esculenta*.

#### Metallothionein

The third most abundant transcript found here had homology to type 2 metallothionein of *Vigna angularis*. Metallothioneins are low molecular (6–7 kD), Cys-rich, metal-binding proteins that have a role in protection against the effects of reactive oxygen species (ROS) by acting as antioxidants as they are potent scavengers of hydroxyl radicals [41,42]. Reactive oxygen species (ROS) may accumulate after the hypersensitive response occurs due to the specific recognition of a pathogen by a plant disease resistance gene and is associated with rapid ion fluxes and protein phosphorylation. ROS may directly repel invading pathogens or serve as signaling molecules that activate defense response [43]. However, ROS resulting from biotic and abiotic stresses can cause cellular damage and need to be detoxified by complex enzymatic and non-enzymatic mechanisms [44].

#### PR Proteins

The reaction between the pathogen elicitor and the R-gene is the first step for an oxidative burst and Systemic Acquired Resistance (SAR). SAR, by its turn, activates gene expression mediated by the master regulator proteinNPR1 (Nonexpressor of pathogenesis-related (PR) genes). NPR1 not only directly induces the *PR *genes but also prepares the cell for secretion of the PR proteins by first making more secretory machinery components [45]. PR (pathogenesis-related) proteins are soluble proteins encoded by a plant host when under attack by a pathogen. They were first described for tobacco [46] and are classified from PR1 to PR10 according to their mobility upon electrophoresis gel. In this work the fourth most found sequences had homology to a PR10 from peanut (Table 2).

#### Cytokinin oxidase-like protein

The fifth most abundant transcripts found here, with 44 clones, had homology to *Arabidopsis thaliana *cytokinin oxidase (Table 2). Cytokinins are essential hormones for plant growth and development. The modulation of cytokinin levels is performed by the irreversible degradation of cytokinins catalyzed by cytokinin-oxydase, [47]. Cytokinin oxydase gene expression has been found to be induced in maize under drought and heat stresses in order to control plant growth under these conditions [47].

### Nodulation-related genes

Nitrogen assimilation is an important process controlling plant growth and development. The assimilation of inorganic nitrogen into carbon skeletons has marked effects on plant productivity, biomass, and crop yield. Inorganic nitrogen is assimilated into the amino acids glutamine, glutamate, asparagine, and aspartate, which serve as important nitrogen carriers in plants. The enzymes involved in the biosynthesis of these nitrogen-carrying aminoacids are glutamine synthetase (GS), glutamate synthase (GOGAT), glutamate dehydrogenase (GDH), aspartate aminotransferase (AAT), and asparagine synthetase (AS) [48]. Each of these enzymes is encoded by a gene family wherein individual members encode distinct isoenzymes that are differentially regulated by environmental stimuli, metabolic control, developmental control, and tissue/cell-type specificity [48]. ESTs with homologies to all of these enzymes were found in this study. In addition, homologues to symbiosis specific genes such as *ENOD*40, Nodulin 35, Nodulin *MtN*21 and nodulation receptor kinases were also found.

### Microsatellites

Molecular markers are useful for genetic map construction, marker-assisted selection in breeding programs, studies of crop evolution, phylogenetic relationships and cultivar protection. For peanut, little variation has been observed with molecular markers, in spite of its considerable phenotypic variability (reviewed by Dwivedi *et al*., 49.). Microsatellite markers have been useful markers in plant genetic research, but they are expensive and labour-intensive to produce. Data-mining microsatellite markers from EST data can be a cost effective option. In the EST sequences published here, 206 microsatellites were found, from which 164 microsatellite markers have been developed and characterized. Almost all microsatellites had low repeat number of di- and tri-nucleotide motifs. Of the di-nucleotide repeats, by far the most common were TC and AT repeats.

In *Arachis*, certain microsatellite types are more polymorphic than others. Dinucleotide repeats are more polymorphic than trinucleotide repeats, AG/TC repeats are more polymorphic than AC/TG repeats, and, for cultivated germplasm, longer microsatellites (15 or more motif repeats) are more polymorphic [17]. The vast majority of microsatellites in ESTs are low repeat number, and accordingly the microsatellite markers developed from these ESTs have low polymorphism in cultivated germplasm (see Additional File 1). Our analysis of microsatellites present in the ESTs and in GSSs shows that longer TC repeats are very rare in both transcribed and non-transcribed DNA, being present in *c*. 0.1% of ESTs, and *c*. 0.2% of genome survey sequences (Figure 2A and 2B). This leads us to believe that unless very large numbers of sequences are produced, the use of microsatellite enrichment strategies [17,50,51] will be the most productive way for cultivated germplasm marker development. In contrast, for wild germplasm the EST microsatellite markers had good levels of polymorphism and have the advantage of being genic. As previously observed, EST microsatellite markers have much potential for work with wild alleles, and for the construction of gene-rich maps [13].

## Conclusion

EST databases provide a great deal of information on the complexities of gene expression patterns, the functions of transcripts and are useful for the development of molecular markers. In this study, EST analysis of the wild relative of peanut, *A. stenosperma *showed that this species has a considerable number of genes related to human health, plant defense, hormone response, all which could be potentially useful for introgression in the cultivated species. To conclude, ESTs produced in this study are a valuable resource for gene discovery, the characterization of new wild alleles, and for marker development.

## Methods

### cDNA libraries construction

*Arachis stenosperma *seeds were germinated in sterile soil. Materials for RNA extraction were collected from three-month old plants: healthy leaves, healthy roots, roots inoculated with 2 mL of a suspension of 10^8 ^cells of *Bradyrhizobium japonicus*, and roots inoculated with 10.000 juveniles (J2) *Meloidogyne arenaria *(Neal) Chitwood race 1. Collected materials were immediately frozen in liquid nitrogen for RNA extraction.

Total RNA was isolated from plant materials using Trizol Reagent (Invitrogen, Carlsbad, CA, USA), according to the manufacture's instructions. The quantity and quality of total RNA was evaluated by spectrophotometry (OD260/280) and formaldehyde-1% agarose gel electrophoresis. Poly (A)^+ ^RNA was extracted from 1 mg of total RNA using the Oligotex Spin Column (Qiagen Inc., Valencia, CA, USA) according to the manufacture's protocol.

Full-length cDNA libraries were constructed using the SMART cDNA synthesis kit in ëTriplEx2 (Clontech, Palo Alto, CA, USA). The resulting cDNA was packed into ë phages using the Gigapack III Gold packaging kit (Stratagene, La Jolla, CA, USA). The pTriplEx2 phagemid clones in *Escherichia coli *were obtained using the mass *in-vivo *excision protocol according to the manufacture's instructions (Clontech, USA). The white clones grown on screening LB medium (Amp/IPTG/X-Gal) were recovered by random colony selection.

### Sequencing and ESTs analysis

Plasmid DNA was isolated from the selected colonies using the alkaline-lysis method and the cDNA inserts sequenced from the 5'-end using specifically designed primer PT2F2 5'-GCGCCATTGTGTTGGTACCC-3'. Sequencing reactions were performed with Big-Dye Terminator Cycle Sequencing Kit, version 3.1 (Applied Biosystems, CA, USA) or DYEnamic ET Terminator Cycle Sequencing Kit (Amersham Pharmacia Biotech) using the Applied Biosystems automated DNA sequencers 3100 and 377.

Base calling and quality assignment of individual bases were done through the use of Phred [52]. Ribosomal, poly(A) tails, low-quality sequences and vector and adapter regions were removed as described by Telles and da Silva [53] with minor adaptations. The resulting sets of cleaned sequences were assembled into clusters of overlapping sequences using the CAP3 assembler [54], with individual base quality and default parameters. Assembled sequences were submitted for comparison against the GenBank database using BLASTx [55] available from the NCBI (*National Center for Biotechnology Information*) [56]. Putative functions of the ESTs were classified according to the Clusters of Orthologous Groups of proteins – KOG [57]. Resistance Gene Analogues (RGAs) were identified in the EST bank by using a BLASTx search against a local database of Arabidopsis NBS encoding genes [58].

### Analysis of microsatellites and development of markers

Microsatellite primers were developed using the module of softwares described by Martins *et al*. [59]. For the analysis, we considered microsatellites with di-, tri-, tetra-, penta- and hexa- nucleotide motifs with six or more motif repetitions. For comparison, microsatellites were also analyzed from clustered *A. hypogaea *transcripts, and *A. duranensis *genome survey sequences (GSSs) submitted by Steven J Knapp to Genbank.

Polymorphism was screened for in the progenitors of a diploid mapping population by PCR. The progenitors of this population are *A. duranensis *K7988 and *A. stenosperma *V10309 [17], both deposited in the Embrapa Genetic Resources and Biotechnology Germplasm Bank. Markers polymorphic for the diploid population were genotyped and map positions determined. For screening for polymorphism in the cultivated peanut, 16 accessions with representatives from all the six botanical varieties were used.

## Authors' contributions

All authors read and approved the final manuscript. KP inoculated plants, constructed libraries, isolated DNA for sequencing, participated in data analysis. SCMLB participated in conceiving the study, inoculation of plants and drafting the manuscript. DJB participated in conceiving the study, SSR marker development, sequence analysis and drafting the manuscript. MCM characterized and mapped SSR markers. FRS analyzed sequences, constructed databank and submitted sequences to Genbank., NFM participated in sequence analysis and performed protein classification. PMG participated in conceiving the study, library construction and drafting the manuscript,

## Supplementary Material

Additional file 1 *Arachis stenosperma *EST-derived Microsatellite markers. Clone name, primer name (a reduced locus name), forward and reverse primers (5' – 3'), repeat motif, repeat type, annealing temperature (Ta), polymorphism for the *A. duranensis *(K7988) × *A. stenosperma *(V10309) cross, linkage groups (LG) in the *Arachis *diploid map (Moretzsohn *et al*. [17], polymorphism for six *A. hypogaea *accessions (A hyp), the number of loci amplified for *A. hypogaea *(# loci), the subjective score for the quality of the amplification products (Score), Top Blastx results, significance of Blastx hits (E-value) and brief comments for the newly developed SSR markers not yet tested. Hyphen (-) means no amplification. Click here for file

## Figures and Tables

**Table 1:** Summary of the *Arachis stenosperma *V10309 EST libraries

***Total number of reads: 8785 clones***	
**Accepted sequences**	*6265 (71.4%)*
**Number of clusters**	*3500*
**Number of contigs**	*963*
**Number of singletons**	*2537*
**Redundancy (%)**	*59.1*
**Homology (% of ESTs) to known sequences**	*55.9*
***Unknown***	*44.1*

**Table 2:** Homologies of the most abundantly expressed RNAs as determined by ESTs redundancy

**# of reads**	**Blast homology**	**Genbank Accession number**	**Best e-value**
115	auxin-repressed protein-like protein (*Manihot esculenta*)	gb|AAX84677.1	6e^-34^
69	Ara h 8 allergen (*Arachis hypogaea*)	gb|AAQ91847.1|	6e^-72^
60	type 2 metallothionein (*Vigna angularis*)	dbj|BAD18379.1|	1e^-16^
56	PR10 protein (*Arachis hypogaea*)	gb|AAU81922.1|	3e-^68^
44	cytokinin oxidase-like protein (*Arabidopsis thaliana*)	emb|CAB79732.1	1e^-120^
39	alcohol dehydrogenase 1; ADH1 (*Lotus corniculatus*)	gb|AAO72531.1|	1e^-114^
38	metallothionein-like protein (*Arachis hypogaea*)	gb|AAO92264.1	1e^-25^
34	proline-rich protein precursor (*Phaseolus vulgaris*)	gb|AAA91037.1	6e^-05^
29	ripening related protein (*Glycine max*)	gb|AAD50376.1	5e^-52^
25	hypothetical protein (*Nicotiana tabacum*)	dbj|BAD83567.1	1e^-38^
